# Neuronal antibodies in pediatric epilepsy: Clinical features and long‐term outcomes of a historical cohort not treated with immunotherapy

**DOI:** 10.1111/epi.13356

**Published:** 2016-03-21

**Authors:** Sukhvir Wright, Ada T. Geerts, Cornelia Maria Jol‐van der Zijde, Leslie Jacobson, Bethan Lang, Patrick Waters, Maarten J. D. van Tol, Hans Stroink, Rinze F. Neuteboom, Oebele F. Brouwer, Angela Vincent

**Affiliations:** ^1^ Nuffield Department of Clinical NeurosciencesJohn Radcliffe University HospitalUniversity of OxfordOxfordUnited Kingdom; ^2^ Department of Pediatric NeurologyErasmus Medical CenterRotterdamThe Netherlands; ^3^ Department of PediatricsLeiden University Medical CenterLeidenThe Netherlands; ^4^ Departments of Pediatric Neurology and NeurologyCanisius HospitalNijmegenThe Netherlands; ^5^ Department of NeurologyUniversity Medical Center GroningenUniversity of GroningenGroningenThe Netherlands

**Keywords:** Autoantibodies, Pediatric epilepsy, Voltage‐gated potassium channel complex, NMDA receptor

## Abstract

**Objective:**

In autoimmune encephalitis the etiologic role of neuronal cell‐surface antibodies is clear; patients diagnosed and treated early have better outcomes. Neuronal antibodies have also been described in patients with pediatric epilepsy without encephalitis. The aim was to assess whether antibody presence had any effect on long‐term outcomes in these patients.

**Methods:**

Patients (n = 178) were recruited between 1988 and 1992 as part of the prospective Dutch Study of Epilepsy in Childhood; none received immunotherapy. Healthy age‐matched bone‐marrow donors served as controls (n = 112). All sera were tested for serum *N*‐methyl‐d‐aspartate receptor (NMDAR), alpha amino‐3‐hydroxy‐5‐methyl‐4‐isoxazolepropionic acid receptor, leucine rich glioma inactivated 1, contactin associated protein like 2 (CASPR2), contactin‐2, glutamic acid decarboxylase, and voltage gated potassium channel (VGKC)‐complex antibodies by standard techniques. No cerebrospinal fluid (CSF) samples were available. Results were correlated with clinical data collected over 15 years.

**Results:**

Seventeen patients (9.5%) were positive for VGKC complex (n = 3), NMDAR (n = 7), CASPR2 (n = 4), and contactin‐2 (n = 3), compared to three (3/112; 2.6%) healthy controls (VGKC complex [n = 1], NMDAR [n = 2]; p = 0.03; Fisher's exact test). Titers were relatively low (≤1:100 for cell‐surface antibodies), but 8 (47%) of the 17 positive samples bound to the surface of live hippocampal neurons consistent with a potential pathogenic antibody. Preexisting cognitive impairment was more frequent in antibody‐positive patients (9/17 vs. 33/161; p = 0.01). Fourteen antibody‐positive patients were treated with standard antiepileptic drugs (AEDs); three (17%) became intractable but this was not different from the 16 (10%) of 161 antibody‐negative patients. In 96 patients with available follow‐up samples at 6 and/or 12 months, 6 of 7 positive antibodies had disappeared and, conversely, antibodies had appeared for the first time in a further 7 patients.

**Significance:**

Neuronal antibodies were found at low levels in 9.5% of patients with new‐onset pediatric epilepsy but did not necessarily persist over time, and the development of antibodies de novo in later samples suggests they could be due to a secondary response to neuronal damage or inflammation. Moreover, as the response to standard AEDs and the long‐term outcome did not differ from those of antibody‐negative pediatric patients, these findings suggest that routine neuronal antibody testing is unlikely to be helpful in pediatric epilepsy. However, the higher incidence of preexisting cognitive problems in the antibody‐positive group, the CASPR2 and contactin‐2 antibodies in 7 of 17 patients, and the binding of 8 of 17 of serum samples to live hippocampal neurons suggest that neuronal antibodies, even if secondary, could contribute to the comorbidities of pediatric epilepsy.

**Key Points**

- Low levels of neuronal antibodies are present in ˜10% of patients with pediatric epilepsy at onset but are not associated with poor long‐term outcomes or treatment intractability
- Antibodies can develop during the course of epilepsy and are not likely to be the sole cause of epilepsy in pediatric patients
- However, if associated with clinical features suggestive of autoimmune encephalitis, this “secondary inflammation” may be immunotherapy responsive as seen in other antibody‐mediated diseases

In adults, autoantibodies to essential neuronal proteins such as the *N*‐methyl‐d‐aspartate receptor (NMDAR) and the voltage gated potassium channel (VGKC)‐complex antigen, leucine rich glioma inactivated 1 (leucine rich glioma inactivated 1 (LGI1), are now widely recognized as an important treatable cause of encephalitis.^1^, ^2^ Patients present with memory loss, confusion, and seizures in limbic encephalitis with predominantly LGI1 antibodies^3^, ^4^ and neuropsychiatric features, movement, and autonomic symptoms in NMDAR‐Ab (antibody) encephalitis.^5^, ^6^ However, the recent characterization of faciobrachial dystonic seizures (FBDS) in patients with LGI1 antibodies has widened the phenotype to include patients presenting with seizure predominance.^7^, ^8^ Recognition of each of these diseases is important, as they are responsive to immunotherapies.

In adult and pediatric patients with epilepsy or seizures without encephalitis, autoantibodies are present in approximately 9–13%.^9^, ^10^, ^11^, ^12^ These patients are more likely to have been classified as “focal epilepsy of unknown cause” and show a tendency toward standard antiepileptic drug (AED) resistance.^11^, ^12^ However, in these studies, follow‐up was short, and immunologic treatments have been tried on an empirical basis at a time when the presence of an antibody was unknown. With increasing interest in the possible etiologic role of autoantibodies in epilepsy, and the recognition that early diagnosis and immunotherapy treatment improves outcome in autoimmune encephalitis, it is important for the clinician to be able to make informed decisions regarding which patients to test and whether the results will affect patient management and epilepsy outcome.^13^, ^14^

Here we studied archived samples from patients who had been sampled within a median 69 days from their first presentation to the neurologist and followed up for many years. None of the patients were given immunotherapies and some were resampled at 6 and 12 months. The results were compared with age‐ and sex‐matched healthy controls.

## Methods

### Patient cohort

Children (aged 1 month to 16 years) were enrolled into the Dutch Study of Epilepsy in Childhood (DSEC) from four participating centers in The Netherlands between 1988 and 1992. Details of exclusion and inclusion criteria have been published previously.^15^, ^16^ Children with a presumed “acute symptomatic” etiology for their epilepsy (defined as seizures occurring only during the first week after the onset of acute neurologic insult, for example, stroke, head trauma, or central nervous system infection, or concurrently with an acute systemic metabolic disturbance, for example, uremia, hyponatremia, or hypoglycemia, or both.^17^) were excluded.

Sufficient volumes of serum were available only in 178 children at enrollment (out of the total cohort of 881 patients who were enrolled and discussed). The median period between the first seizure and first blood sampling was 69 days (range 0 days to 6.4 years). All of the serum samples were stored at −20°C from collection. A previous study by the DSEC found no significant differences in sex, etiology, or epilepsy syndrome between those children with available samples and those without.^18^ Follow‐up serum samples from 96 patients taken at 6 months (n = 30), 12 months (n = 34), and 6 and 12 months (n = 32) after intake were also available for testing. In addition, 112 age and sex‐matched control samples came from age‐matched sibling donors of bone marrow transplantations (BMTs), collected between 1985 and 1995 and stored under the same condition as the patients' sera.

### Ethics

The DSEC was approved by the ethics committees of all involved hospitals, and informed consent was obtained in all cases before enrollment.^15^, ^19^

### Epilepsy classification and definitions

At enrollment, classification of the seizures and epilepsy syndromes was made after discussion by three participating pediatric neurologists according to the 1989 International League Against Epilepsy (ILAE) criteria. This was revised after 2 years and at the end of the most recent intractability study,^20^ as some children proved to have neurologic brain‐related morbidities. In view of the new terminology published in the most recent reorganization of the ILAE seizure and epilepsy classification, and to facilitate interpretation of the new serologic data with the preexisting clinical data, we have provided definitions of the terms used in Table 1.

**Table 1:** Definitions of terminology used from the preexisting historical clinical database of the Dutch Study of Epilepsy in Childhood

Terminology	Definition
Idiopathic epilepsy (IE)	Epileptic syndromes with particular clinical characteristics and specific EEG findings. Unknown origin but presumed genetic etiology
Remote symptomatic epilepsy (RSE)	Epilepsies considered the consequence of a known or suspected disorder of the central nervous system resulting in a static encephalopathy. All children with mental retardation (MR) with epilepsy of unknown cause were classified as RSE
Cryptogenic epilepsy (CE)	Epilepsies of unknown origin that do not conform to the criteria for IE or RSE
Terminal remission	Interval between the very last seizure and the end of follow‐up
Fast response to medication	6 Months of remission starting within 2 months after initiation of AED
Intractability	No remission exceeding 3 months (at least one seizure per 3 months) during a minimum period of 1 year of observation despite adequate treatment.^16^ (Arts et al). Early onset intractability: onset of intractability within the first 5 years of follow‐up. Late onset intractability: onset after the first 5 years of follow‐up

EEG, electroencephalogram; AED, antiepileptic drug.

### Antibody testing of serum samples

Antibodies to the VGKC complex and to the intracellular enzyme glutamic acid decarboxylase (GAD) were measured by radioimmunoassays. To avoid reporting results of low specificity,^21^, ^22^ VGKC‐complex antibody positivity was set at >400 pm and GAD positivity at >100 units/ml.^11^ Cell‐based assays (CBAs) were used to detect antibodies to the NMDAR, alpha amino‐3‐hydroxy‐5‐methyl‐4‐isoxazolepropionic acid receptor (AMPAR), LGI1, CASPR2, and contactin‐2. These tests were scored on a visual scale: 0, no binding; 1, low but specific binding; to 4, strong binding to all transfected cells by two independent observers as previously described^3^, ^5^ and in use for routine diagnostics. All positive samples were confirmed, and then tested blind by LW as part of a routine antibody service, with dilutions to assess the titer; the positives samples were also tested for binding to the surface of live hippocampal neurons in culture, prepared from P0 Sprague‐Dawley rat pups as described previously.^23^, ^24^

### Statistical analysis

Descriptive statistics were used to summarize patient data. Fisher's exact test was used to compare categorical data. Data were analyzed using GraphPad Prism 6.0.

## Results

### Autoantibody testing

**Figure 1:**
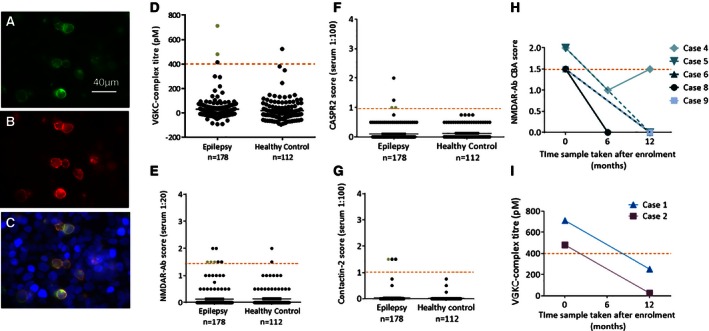
Autoantibody testing results of the epilepsy and healthy control cohorts. The transfected CASPR2‐EGFP tagged transfected HEK cells (green) are shown (**A**). Serum from patient 16 binds to the surface of the CASPR2 transfected cells, seen with anti‐human IgG labelling (red, **B**).The transfected cells (**A**) and anti‐human immunoglobulin (IgG)–labelled cells (**B**) colocalize indicating a positive result for this patient (**C**). The scatter diagrams show the titers and CBA scores of positive tests for each antigen tested at the onset of epilepsy compared with healthy controls; VGKC complex (**D**), NMDAR (**E**), CASPR2 (**F**), and contactin‐2 (**G**). The red dashed line indicates the positive cut‐off used for each assay. The serum samples highlighted by green dots were positive on surface hippocampal staining in vitro. When available, follow‐up samples were tested; four of five NMDAR‐Ab positive patients and both VGKC‐complex antibody‐positive patients were negative at either 6 or 12 months after intake (**H**,** I**). Patient 4 showed an initial reduction in antibody levels then increase over time. These fluctuating antibody levels did not correlate with developmental regression or seizure activity.

Seventeen patients (17/178; 9.5%) were positive for one antibody (VGKC complex [n = 3]; NMDAR [n = 7], CASPR2 [n = 4], and contactin‐2 [n = 3], compared to 3 [2.6%] of the 112 healthy controls (VGKC complex [n = 1], NMDAR [n = 2]; p = 0.03; Fisher's exact test; see Fig. 1, Table 2). Antibodies to LGI1, AMPAR, or GAD were not identified in any patients or controls. Although antibodies binding to the cell‐surface antigens were not high (scoring 1 at dilution 1:20 in 5 and at dilution 1:100 in 9), eight of these samples bound to the surface of live hippocampal neurons (see Table 2), which suggests potential clinical relevance.

**Table 2:** Demographic, clinical, and paraclinical features, and long‐term outcomes of antibody‐positive epilepsy patients

Case no./timing of sample (from onset of epilepsy, days)	Age and sex	Seizure type at onset	Type of epilepsy/etiology	Associated clinical features	Treatment history first 5 years/at end of follow‐up (FU, years)	Epilepsy course/seizure type at end of 5 year follow‐up/outcome (TR, years)	Ab positivity (pm/titers)
1 (33)	4 F	CPS	Localization related symptomatic/remote symptomatic	Right hemiplegia Learning difficulties	4 AEDS: no fast response/off AED at final FU (14.6)	Improving course SPS, SE, unclear seizures TR = 10.2	VGKC (712 pm)^a^ 252 pm at 12 months
2 (0)	1.7 M	TC	Localization‐related symptomatic/cryptogenic	Mental retardation, not progressive	3 AEDs: no fast response/on AED at final contact (14.7)	Poor course TC, SPS, CPS, unclear seizures TR < 1 year, intractable	VGKC (480 pm)^a^ 25 pm at 12 months
3 (2,333)	7.4 F	TC	Generalized idiopathic/idiopathic	Mild developmental delay	No AEDs; not on AED at final contact (15.3)	Improving course TC, unclear seizures TR = 1.5	VGKC (414 pm)
4 (365)	7.4 M	Absences	Generalized idiopathic childhood absence epilepsy/idiopathic	–	2 AEDs: fast response/off AED at final contact (15.7)	Good course absences TR = 15.6	NMDAR (1 in 100) 1 in 20 at 12 months
5 (106)	1.5 F	Minor motor and absences	Generalized idiopathic childhood absence epilepsy/idiopathic	–	2 AEDs: no response (bad compliance)/on AED at final contact (13.5)	Improving course Minor motor and absences TR = 1.7	NMDAR (1 in 100) Negative at 12 months
6 (4)	12.6 F	TC	Generalized idiopathic with photosensitivity/remote symptomatic	Mild learning difficulties (LD) –attended regular school	1 AED: fast response/on AED at final contact (13.1)	Good course TC TR = 13.1	NMDAR (1 in 20) Negative at 12 months
7 (0)	12 M	SE	Localization related symptomatic/remote symptomatic	Learning difficulties, IQ < 50 Tetraparesis	2 AEDs: no fast response/on AED at death (died 1.4 years after enrollment)	SE, unclear small seizures TR = 1	NMDAR (1 in 20)^a^
8 (11)	3.6 F	Atonic seizures	Generalized Lennox‐Gastaut syndrome/remote symptomatic	Mild global delay	2 AEDs: no fast response/on AED at final FU (12.7)	Deteriorating course Atonic seizures, TC TR = 0.5	NMDAR (1 in 20)^a^ Negative at 6 months
9 (89)	6.6 F	Absences	CAE/idiopathic	–	1 AED: fast response/off AED at final FU (14.8)	Good course Absences TR = 14.8	NMDAR (1 in 20)^a^ Negative at 12 months
10 (0)	15.5 F	TC	IGE/idiopathic	–	No AED/off AED at final FU (2)	Lost to follow‐up after 2 years TC TR = 2	NMDAR (1 in 20)
11 (46)	4.1 M	SE	Localization related symptomatic/RS including MR	Mild learning difficulties, autism spectrum disorders	1 AED: fast response/off AED at final FU (15.9)	Good course SE TR 12.1	Contactin‐2 (1 in 100)^a^
12 (124)	4.2 M	Unclear seizures	Remote symptomatic including MR	Global mental retardation, spasticity, visual problems	1 AED: fast response/on AED at final FU (5)	Improving course TC with focal onset TR > 2	Contactin‐2 (1 in 100)
13 (271)	8.8 F	Atonic, astatic	BECTS/idiopathic	–	3 AED: no fast response/off AED at final FU (5)	Improving course SPS with generalization TR > 2	Contactin‐2 (1 in 100)
14 (31)	12.5 M	SPS	Benign partial epilepsy/idiopathic	–	Never used AED	Good course SPS with generalization TR > 5	CASPR2 (1 in 100)^a^
15 (49)	8.9 M	TCS	IGE/idiopathic	–	1 AED: fast response/no AED at final FU (14)	Good course TCS TR > 5	CASPR2 (1 in 100)^a^
16 (1)	10.5 M	PS with secondary generalization	Localization related cryptogenic/cryptogenic	–	3 AED: no fast response, polytherapy/on AED at final FU (13.3)	Poor course, intractable Clustered PS with gen leading to hospitalization TR < 0.1	CASPR2 (1 in 100)
17 (130)	0.6 M	CPS, myoclonic, atonic	Secondary generalized multifocal with atonic and atypical absence seizures/RS including MR	Severe mental retardation Febrile seizures during FU	4 AED: no fast response/on AED at final FU (16.5)	Improving course. Intractable at 5 years – bad compliance. TCS TR > 5	CASPR2 (>1 in 100)

AED, antiepileptic drug; BECTS, benign epilepsy with centrotemporal spikes; CAE, childhood absence epilepsy; CPS, complex partial seizures; CT, computed tomography; EEG, electroencephalography; FU, follow‐up; IGE, idiopathic generalized epilepsy; MJ, myoclonic jerks; PS, partial seizure; RS, remote symptomatic; SPS, simple partial seizures; SWD, spike‐wave discharge; TC, tonic–clonic; TCS, tonic–clonic seizure; TR, terminal remission; SE, status epileptic.

a Positive serum staining on the surface of hippocampal neurons in vitro.

### Clinical features of antibody positive patients

The clinical and paraclinical features, treatment responses, and outcomes of the antibody‐positive patients are listed in Table 2. All three patients with VGKC complex Abs had cognitive impairment/learning difficulties (this was not present in the VGKC complex antibody‐positive healthy control). The two patients (cases 1 and 2; Table 2) with the highest titers (712, 480 pm) had focal epilepsy that was difficult to treat, needing at least three AEDs for seizure control initially. Although there were no antibodies detected to the VGKC‐complex‐associated proteins, LGI1 and CASPR2 in these samples, both bound to the surface of hippocampal neurons suggesting potential clinical relevance.

Three of the seven NMDAR‐Ab‐positive patients had learning difficulties before the onset of epilepsy (cases 6–8), including one severely affected (case 7), and required AEDs until the end of follow‐up. Three others (cases 4, 5, and 9) had childhood absence epilepsy with 3‐Hz generalized spike‐wave discharge on electroencephalography (EEG). Three patients were positive for contactin‐2 antibodies (cases 11–13); two were known to be on the autistic spectrum and one had pharmacoresistant benign epilepsy with centrotemporal spikes (BECTS) requiring three AEDs for seizure control. Four patients were positive for CASPR2 antibodies; two (cases 16, 17) had focal epilepsy with periods of resistance to AEDs.

### Comparison of clinical features and long‐term outcomes between antibody‐positive and antibody‐negative patients

There were no differences in the sex distribution or age at onset of epilepsy between antibody‐positive and antibody‐negative patients. Seizure semiology and frequency also showed no long‐term difference, and computed tomography (CT) and EEG findings were comparable (Table 3). Overall, however, there was a significantly higher rate of cognitive impairment/developmental delay in the antibody‐positive patient group (9/17 vs. 33/161; p = 0.01). These features were all present before the onset of epilepsy (and hence antibody testing), and included patients with structural brain abnormalities, mild autism, and severe global development delay.

**Table 3:** Comparison of clinical features and outcomes of new onset epilepsy antibody positive and negative patients

Characteristic	Antibody positive (n = 17)		Antibody negative (n = 161)		p‐Value
Sex	M:F – 9:8		M:F – 72:89		
Median age of presentation	5.7 years (range 0.9–15.5)		6.2 (range 0.2–15.8)		
Type of epilepsy at enrollment	Generalized	9 (53%)	Generalized	75 (47%)	0.8
	Focal	6 (35%)	Focal	82 (51%)	0.3
	Other	2 (12%)	Other	4 (2%)	0.1
Frequency of seizures within first 6 months	1–3	6 (35%)	1–3	71 (44%)	0.6
	4–25	4 (24%)	4–25	34 (21%)	0.8
	Uncountable	7 (41%)	Uncountable	56 (35%)	0.6
Etiology at onset	Idiopathic	8 (47%)	Idiopathic	87 (54%)	0.6
	RS incl MR	7 (41%)	RS incl MR	43 (27%)	0.3
	Cryptogenic	2 (12%)	Cryptogenic	31 (19%)	0.7
Preexisting neurologic signs/abnormal neurologic examination	3 (17.6%)		17 (10.6%)		0.4
Mental retardation/cognitive impairment at intake	9 (52.9%)		33 (20.4%)		0.01^a^
History of febrile seizures before or after intake	1(5.8%)		32 (19.8%)		0.2
Family history	2 (11.7%)		20 (12.4%)		1
Status epilepticus as presenting feature	2 (11.7%)		9 (5.6%)		0.2
Abnormal EEG at intake	14 (82%)		126 (78%)		1
CT at intake	Normal	8 (47%)	Normal	85 (53%)	0.8
	Abnormal	4 (24%)	Abnormal	29 (18%)	0.5
	Not done	5 (29%)	Not done	48 (30%)	1
Polytherapy during FU, range (2–16 years)	4/14 (28.5%)		22/143 (15.4%)		0.3
On AED at final contact, range (2–16 years)	8/14 (57%)		44/143 (30.7%)		0.07
Intractable at last contact	3 (2 with late onset) (17.6%)		16 (8 with late onset) (9.9%)		0.4

AED, antiepileptic drug; MR, mental retardation; RS, remote symptomatic.

a Analyzed by Fisher's exact test.

At 5‐year follow‐up, 65% (11/17) of patients in the antibody‐positive and 78% (125/161) in the antibody negative group had been seizure‐free for >12 months (not significant). At final contact, 57% (8/14) of the antibody‐positive patients were taking AEDs compared to 31% (44/140) of antibody‐negative patients (p = 0.07, Table 3), but the rate of intractability was not different between the two groups (p = 0.18, Fisher's exact test).

### Testing of follow‐up samples

Of the 17 patients who were antibody positive at intake, further samples at 6 and 12 months were available for antibody testing in 7 (Fig. 1H,I). Changes in these short‐term antibody levels (Fig. 1H,I) did not correlate with changes in seizure frequency or cognitive development (Table 2). Moreover, 7 (7.7%) of 89 sera from patients who had been antibody negative (n = 161) were found to be positive at follow‐up (NMDAR‐Abs [n = 3], CASPR2‐Abs [n = 2], CASPR2‐ and NMDAR‐Abs [n = 1], and contactin‐2‐Abs [n = 1]). Two of these patients became intractable in the long‐term (cases 27 and 64). The antibodies and clinical features of these patients are listed in Table S1.

## Discussion

Autoantibodies to neuronal surface antigens have been reported in adults and children with epilepsy and could indicate an immune basis with obvious management implications.^11^, ^12^, ^13^ However, these studies have been complicated by some use of immunotherapies, and long‐term treatment and outcome data of untreated antibody‐positive patients have not been reported. We were able to test a large, historical cohort of patients with pediatric epilepsy for neuronal surface antibodies and relate the findings to their epilepsy course over time. The frequency of antibodies were similar to those reported previously, but were mainly transient and occurred sporadically; despite lack of immunotherapies, most autoantibody positive patients had a good outcome and responded to standard AEDs. The results suggest, therefore, that routine antibody testing is not necessarily helpful in children with epilepsy and should be restricted to those with evidence of neuroinflammatory disease. Nevertheless, the number of patients (n = 11) who had or developed antibodies to CASPR2 or contactin‐2, proteins that are linked to genetic forms of epilepsy or neurodevelopmental disorders, is an intriguing finding that deserves further study.

The frequency of antibody positivity in this study cohort (9.5% compared with 2.6% in controls) was similar to that published previously for both adult and pediatric epilepsies (10–16% in patients and <5% in controls). Patients with acute symptomatic epilepsy had been excluded and, importantly, the patients did not have any associated clinical features of encephalitis (e.g., confusion, memory loss) at the time of sampling, and none had received immunotherapies. All cell‐based assay–positive samples obtained at onset contained relatively low antibody titers, 8/14 bound to the surface of hippocampal neurons in vitro, and most available follow‐up samples had normalized by 12 months, whereas antibodies had appeared de novo in seven patients. Although samples were taken as close to seizure onset as possible, the median sampling time of 69 days means that some were taken after the establishment of epilepsy and the results could, therefore, reflect the consequences of the epileptogenic process rather than being the primary cause.

Indeed, low‐titer antibodies to VGKC complex or NMDARs have been found in some patients without autoimmune neurologic diseases,^21^, ^25^ and the fact that NMDARs were found in some children with generalized absence epilepsy makes them unlikely to be pathogenic in these cases. However, the binding to the surface of live hippocampal neurons, present in 8 of 17 sera, suggested that the antibodies could be pathogenic if they reach the brain parenchyma. In the remaining patients whose antibodies did not bind to the neurons or to LGI1, the VGKC‐complex Abs may be markers for a neuroinflammatory process rather than for an antibody‐mediated syndrome, as recently discussed.^12^, ^22^, ^26^ Surprisingly, therefore, antibodies to CASPR2 or contactin‐2, the other known components of the VGKC complex, were found in 11 pediatric epilepsy patients (seven at onset, four during follow‐up), but not in controls. This finding draws attention to the growing area of shared targets and partially overlapping phenotypes between antibody and genetic forms of pediatric neurologic disease.^12^, ^27^

Despite the limitations of a retrospective study with no access to cerebrospinal fluid (CSF) samples for testing, this observational study enabled us to investigate the long‐term outcome of antibody positivity in a cohort of patients with pediatric epilepsy in whom immunotherapies were not used. Because most patients responded well to standard AEDs and had a good long‐term outcome, this questions the pathologic relevance of these low positive antibodies particularly as, in most cases, the antibodies were transient and disappeared by 6 or 12 months follow‐up. It seems likely that the antibodies may have been a secondary response to neuronal damage before the seizures came under control, rather than the primary pathogenic agent. Nevertheless, this is not unprecedented and the development of high levels of NMDAR‐Abs following herpes simplex virus encephalitis (HSVE) with neurologic deterioration (movement disorder, behavioral change, seizures, and worsening of brain lesions on magnetic resonance imaging [MRI]), in patients with no evidence of reactivation of the herpes simplex virus,^28^ and their response to immunotherapy,^29^ demonstrates that this “secondary inflammation” may still be pathogenic and respond to immunotherapy.

From these results, a “mono‐immunogenic” cause for pediatric epilepsy is unlikely, with neuronal antibodies forming only part of the complex etiologic framework that includes inflammation, structural abnormalities, and genetic susceptibility; similar diverse factors may explain the final outcomes. Future studies investigating predictive biomarkers in epilepsy need to include all these factors to indicate the likely progression of disease and treatment response. This would be invaluable to both patients and clinicians,^30^ particularly in drug‐resistant refractory cases with associated comorbidities, commonly seen in pediatric epilepsy. This group of patients may benefit most from further studies into antibody presence, antigenic targets, relevance, and immunotherapy treatment trials.

## Disclosure of Conflict of Interest

AV, BL, PW, and the Nuffield Department of Clinical Neurosciences in Oxford receive royalties and payments for antibody assays. The remaining authors have no conflicts of interest. We confirm that we have read the Journal's position on issues involved in ethical publication and affirm that this report is consistent with those guidelines.

## Supporting information


**Table S1.** Clinical features of latent antibody‐positive epilepsy patients.
