# Supplementary material for: Neuronal antibodies in pediatric epilepsy: Clinical features and long‐term outcomes of a historical cohort not treated with immunotherapy
Source: Epilepsia. 2016 Mar 21;57(5):823–31. doi: 10.1111/epi.13356 (PMC4864754; doi:10.1111/epi.13356)
Supplement: Supplementary file 1 — Table S1. Clinical features of latent antibody‐positive epilepsy patients. [file EPI-57-823-s001.docx]

| Patient | Antibody positivity at: | | | Clinical and paraclinical features | Epilepsy course/outcome |
| --- | --- | --- | --- | --- | --- |
|  | Intake | 6 months | 12 months |  |  |
| 131 | negative | **NMDAR-Ab**  **Positive**  **(1 in 100)**  **CASPR2 positive (1 in 100)** | negative | 1.5 yr Male  Before intake tonic-clonic and absence seizure varying in intensity  Abnormal EEG at intake, normal EEG after partial sleep deprivation, normal EEG at 6 months. Normal CT | No further seizures at 5 year F/U  2 AEDs, monotherapy, fast response  Total FU 15.1 years  TR 15.1 years, not intractable |
| 136 | negative | No sample | **NMDAR-Ab Positive**  **(1 in 500)** | 0.5 yr Female  Before intake, clusters of spasms 1/day Tuberous sclerosis, West syndrome, LD  Abnormal EEG at intake (hypsarrythmia), and at 6 months  Abnormal CT scan | After intake, several absence like events per day during period of one year  1 AED used, no fast response  Total FU 13.7 yrs  TR 1.5 years, not intractable |
| 27 | negative | No sample | **NMDAR-Ab**  **Low positive**  **(1 in 20)** | 1.3 yr Male  Before intake, 15 febrile convulsions clusters  Behavioural problems, no learning difficulties , positive family history  Normal EEGs at intake and 6 months  Normal CT | During FU, monthly clusters of febrile seizuress, absences, CPS 1-6/week.  2 periods intractability >1 yr in first 5 yr FU  Total FU 13.9 yrs  TR 0, intractable at endpoint |
| 124 | negative | **NMDAR-Ab Low positive**  **(1 in 20)** | negative | 4.2 yrs Male  Before intake, 2 unclear seizures and febrile convulsions  Normal EEG at intake, abnormal at 6 months. Normal CT | After intake, 5 seizures (type unknown) within 2 months  One AED used, fast response  Total FU 14.5 yrs,  TR 14.3 yrs, not intractable |
| 64 | negative | **CASPR2 positive (1 in100)** | No sample | 3.4 yrs male  Innumerable seizures starting from a few days after birth, multiple times/day  EEG : consistent with BECTS  Daily multiple CPS  Hypothalamic hamartoma discovered MRI at 5 year FU; surgery without success | During FU 2-6 seizures/day  gelastic seizures, CPS  Total FU 14 years  TR= 0 yrs  Intractable |
| 152 | negative | **negative** | **CASPR2 positive (1 in100)** | 7.5yr female  1-3 fits before intake (type of seizures: 1 CPS and 2 CPS with generalization), normal neurological exam  Abnormal EEG with epileptic abnormalities during hyperventilation and intermittent photic stimulation, normal CT  No retardation | Infrequent seizures after intake  Total FU 15.9 yrs  TR = 4 yrs |
| 104 | negative | **negative** | **contactin-2 positive (1 in 100)** | 6.9 yrs female  2-20 absences per day  Abnormal EEG with 3Hz spike and wave ; CT not done  No retardation | Seizures after intake: 1-2x/day absences or CPS during 2 months after intake. Eleven months later, 2 CPS within 4 days.  Total FU=14.7 yrs  TR=12.6 yrs |

**Supplementary Table 1 Clinical features of latent antibody positive epilepsy patients**

Abbreviations: AED anti-epileptic drug, BECTS Benign Epilepsy with Centrotemporal Spikes, CPS complex partial seizure, CT computed topography, EEG electroencephalogram, FU follow-up, TR terminal remission.
